# The singularities of light: intensity, phase, polarisation

**DOI:** 10.1038/s41377-023-01270-8

**Published:** 2023-09-19

**Authors:** Michael V. Berry

**Affiliations:** H H Wills Physics Laboratory, Bristol, BS8 1TL UK

**Keywords:** Optics and photonics, Optical physics

## Abstract

In modern optics, light can be described at different levels: as rays, as scalar waves, as vector fields, and as quantum fields. In the first three levels, there are singularities—characteristic features, useful in interpreting phenomena at that level. In geometrical optics, the singularities are ray caustics; in scalar wave optics, they are phase singularities (=wave dislocations= wave vortices = nodal manifolds); in vector waves, they are singularities where the polarisation of light is purely linear or purely circular. The singularities at each level are dissolved at the next level. Similar singularities occur in all waves, not just light.

*Marking 50 years since the discovery, in collaboration with J.F. Nye, of phase singularities as a general phenomenon in wave physics*.

## Introduction

Geometry dominates modern optics, in particular through understanding light in terms of its singularities. From this perspective, there are different levels of description in optics, each characterised by different singularities. Analogous considerations apply to other types of wave: quantum, acoustic, elastic, water…. In this review, three qualitatively different singularities will be described. The emphasis will be on singularites that are *natural*, in the sense that they are stable under perturbation; equivalent terms for this kind of naturalness are typicality, genericity, structural stability, and universality.

In geometrical optics (see the section ‘Rays’), the singularities are caustics: envelopes of families of rays. In scalar wave optics (see the section ‘Phase’), there are phase singularities, also called wave vortices, wavefront dislocations, and nodal lines (in 3D). In electromagnetic (i.e. vector) waves (see the section ‘Polarisation’), there are singularities of polarisation: lines (in 3D) on which waves are purely circularly polarised or purely linearly polarised.

This review includes some personal remarks.

## Rays

The coarsest level is geometrical optics, in which light fields are described by families of rays. Here the singularities are caustics: focal lines and surfaces, that is, the envelopes of ray families, on which the intensity diverges; therefore caustics are the singularities of bright light. These singularities are classified by the mathematics of catastrophe theory^1–4^, providing a list of the geometric forms of caustics that are stable under generic perturbations.

Many phenomena are described by caustics: rainbows^5^; the bright lines of focused sunlight on the bottoms of swimming-pools; bright-edged shadows of floating insects^6^; twinkling starlight^7,8^ (whose statistics involve a competition between singularities^9^); mirages (the subject of an interesting misunderstanding^10^); unusual colours inside prisms^11^; beams that bend in free space^12–14^; and distorted images in curved mirrors^15,16^ and gravitational lensing^17,18^.

## Phase

In wave optics, the caustic singularities of families of rays are smoothed by diffraction, which decorates them with rich and ubiquitous interference patterns^19,20^, described by a new class of special functions (‘diffraction catastrophes’^21^), represented by oscillatory integrals (chapter 36 of ref. ^22^ and refs. ^23,24^). The wave decorations exhibit interesting scaling laws, with implications for gravitational lensing^25^. In white light, caustics display interesting colours when diffraction is incorporated^26,27^.

Wave optics, when represented by complex scalar wavefunctions, introduces the additional concept of phase, which has its own singularities. Equivalent terms for phase singularities are optical vortices, nodal manifolds or wavefront dislocations^28,29^. On phase singularities, the light intensity is zero, so these are the singularities of dark light. Geometrically, they are lines in space, or points in the plane, around which the phase changes by a multiple of 2*π* (generically ±1) and the phase gradient vector circulates—hence the term optical vortices.

Phase singularities are complementary to caustics, not only because the former are dark (zero intensity) and the latter are bright (infinite intensity), but also in the sense of Niels Bohr: caustics are prominent features in the short-wave asymptotic regime, in which phase singularities are too close to be clearly resolved—because these are fine-scale features, clearly discernable only in the opposite case of high magnification, where caustics are smoothed out and so are no longer distinct features.

The darkness of a phase singularity can be regarded as perfect descructive interference^30^, underlying several interesting features. In white-light interference, universal colour patterns inhabit the near-darkness^31,32^. And because the phase changes by a multiple of 2π around a singularity, the local phase gradient vector rises to infinitely large values there. Therefore phase singularities are powerful sources of superoscillations (band-limited functions that vary faster than their fastest Fourier component)^33^, with many applications^34,35^, for example to sub-wavelength microscopy^36–39^ and in mathematics^40^. The optical applications of superoscillations depend on a fundamental fact: there is a diffraction limit for bright light, but there is no such limit for dark light (evoking the mathematician André Weil’s playful “…principle of anti-inteference, which would have light burst forth from two darknesses.”^41^).

Phase singularities can form intricate patterns, for example as fine detail in diffraction catastrophes^19,20,42^ and near spiral phase plates^43,44^. They can organise the coloured interference patterns formed by white light^31,32,45^. They occur in all types of quantum^46–52^ or classical (e.g. acoustic^53^ and tide^54–58^) waves and have been extensively reviewed^59–61^. In three dimensions, phase singularity lines can be linked and knotted^62–67^.

There has been a confusion in which wave vortices are regarded as inevitably associated with orbital angular momentum (OAM). The association holds for the simplest cases, e.g. optical beams that are eigenstates of OAM, but in general the concepts are distinct, as counterexamples demonstrate^68^. We are celebrating 30 years since the discovery of OAM as a practical resource in optics^69^. I remember L. Allen excitedly explaining OAM to me during a train journey in the early 1990s^70^.

## Polarisation

Incorporating the vector (electromagnetic) nature of light brings further singularities, corresponding to the new physical property thus introduced, namely polarisation. In electromagnetic waves, the polarisation singularities that are stable under perturbations are lines in three dimensions. There are two types^71–75^: C singularities, on which the polarisation is purely circular, and L singularities, on which the polarisation is purely linear.

In direction space, polarisation singularities play a central role in crystal optics^76–81^ (notably conical refraction^82,83^), and in the pattern of polarised light in the blue sky^84^. The C and L lines are different for the electric and magnetic fields^74^, but coincide for paraxial fields^85^. In the presence of optical absorption (or gain) important singularities are the degeneracies of nonhermitian matrices^86–89^.

Polarisation singularities were discovered by J.F. Nye and his student J.V. Hajnal. Nye was an original scientist in several areas^90^; he was my only significant senior collaborator, with a positive influence on my scientific development, beginning with our paper on phase singularities^28^. Nye’s book^29^ is an excellent account of optical singularities, and an excellent complementary description is the book by Gbur^91^.

## Concluding remarks

Historically, all three levels of singularity can be considered to have originated (in separate discoveries) in the same decade: the 1830s^92^.

As well as representing physics at each level, these optical and wave geometries illustrate the idea of asymptotically emergent phenomena^93,94^. The levels form a hierarchy, with each deeper level of theory eliminating the earlier singularities and generating new ones. Thus, the caustic singularities of ray theory are softened by scalar wave theory, enabling us to see the interference decorations, in the form of diffraction catastrophes. Interference fringes near caustics are not only brighter than elsewhere; they are also more widely separated—for smooth caustics, O(wavelength^2/3^) rather than O(wavelength). That is why our unaided eyes can see the wave features of light, vastly magnified in the sky, as the Airy supernumerary fringes decorating rainbows (ref. ^5^, and see the section ‘Rays’ of ref. ^95^). And the phase singularites introduced into scalar wave theory are themselves dissolved by the vector nature of light and replaced by the C and L polarisation singularities.

The hierarchy approach has predictive power, pointing towards the future of optical singularity theory. The phase singularities of scalar light can be regarded as windows, through which can be glimpsed the faint glimmering of the quantum vacuum (or, near acoustic vortices, the whispering of Brownian motion). Therefore phase singularities will have quantum cores^96,97^—a connection with quantum optics and, more fundamentally, quantum field theory, yet to be explored in detail. Related to this is the prediction of large momentum transfers to small particles^98^ located near phase singularities.

An extension of the hierarchy idea would be to regard polarisation singularites as windows to the deeper level of quantum optics; perhaps the counting statistics of photons emitted by excited atoms would be modified near C and L singularities. This too is unexplored territory. More fundamentally, one can speculate about possible new singularities in the quantum field theory of light; these would be windows down to a deeper and as yet unimagined level of our understanding of light, beyond quantum.
